# Therapeutic impact of dietary vitamin D supplementation for preventing right ventricular remodeling and improving survival in pulmonary hypertension

**DOI:** 10.1371/journal.pone.0180615

**Published:** 2017-07-07

**Authors:** Hiroaki Tanaka, Masaharu Kataoka, Sarasa Isobe, Tsunehisa Yamamoto, Kohsuke Shirakawa, Jin Endo, Toru Satoh, Yoji Hakamata, Eiji Kobayashi, Motoaki Sano, Keiichi Fukuda

**Affiliations:** 1 Department of Cardiology, Keio University School of Medicine, Tokyo, Japan; 2 Actelion Pharmaceuticals Japan Ltd., Tokyo, Japan; 3 Department of Cardiology, Kyorin University School of Medicine, Tokyo, Japan; 4 Department of Basic Science, School of Veterinary Nursing and Technology, Faculty of Veterinary Science, Nippon Veterinary and Life Science University, Tokyo, Japan; 5 Department of Organ Fabrication, Keio University School of Medicine, Tokyo, Japan; Niigata Daigaku, JAPAN

## Abstract

**Background:**

Pulmonary hypertension (PH), caused by elevated pulmonary vascular resistance, leads to right heart failure and ultimately death. Vitamin D deficiency can predispose individuals to hypertension and left ventricular dysfunction; however, it remains unknown how serum vitamin D level is related to PH and right ventricular (RV) dysfunction.

**Methods:**

Serum 25-hydroxyvitamin D [25(OH)D] levels were assessed in PH patients for an association with disease severity. To examine whether vitamin D supplementation could prevent the development of pulmonary vascular remodeling and RV dysfunction in PH, a rat model of PH was fed either normal chow or a high vitamin D diet.

**Results:**

The majority (95.1%) of PH patients had 25(OH)D levels in the insufficiency range, which is associated with increased mean pulmonary artery pressure, increased pulmonary vascular resistance, and decreased cardiac output in PH patients. Vitamin D supplementation significantly increased serum 25(OH)D levels and improved survival in PH rats. Interestingly, while the supplemented rats retained the typical increases in medial thickness of the muscular pulmonary arteries and RV systolic pressure, RV cardiomyocyte hypertrophy and B-type natriuretic peptide expression was significantly attenuated.

**Conclusions:**

Vitamin D deficiency is frequently seen in patients diagnosed with PH and low serum levels of 25(OH)D are associated with severity of PH and RV dysfunction. Vitamin D supplementation in PH rats improved survival via ameliorating pathological RV hypertrophy. These findings suggest an insufficient intake of vitamin D might potentially accelerate RV dysfunction, leading to a crucial clinical impact of vitamin D supplementation in PH.

## Introduction

Pulmonary hypertension (PH) is a severe disease characterized by remodeling of the pulmonary vessels and leading to a persistent increase in pulmonary vascular resistance (PVR) and right ventricular (RV) remodeling, ultimately resulting in RV failure and death. While recent advances in the management of PH have led to reduced morbidity and mortality events [1,2], PH remains progressive and fatal [3,4], highlighting the need for improved treatment and prevention of disease progression.

Vitamin D, known as cholecalciferol in animals and ergocalciferol in plants, is a steroid hormone mainly acquired through production in response to sunlight exposure or by intake from dietary sources. Vitamin D is biologically inactive and requires two hydroxylation steps, first to 25-hydroxyvitamin D (25(OH)D) in the liver followed by formation of the active metabolite 1,25-dihydroxyvitamin D; the latter metabolism occurs primarily in the kidney and to a lesser extent in most other tissues. It is now recognized that vitamin D plays an important role in mineral homeostasis and the skeletal system, as well as in the blood vessels and heart [5,6]. Epidemiological studies also suggest a relationship between vitamin D and hypertension, cardiovascular events, myocardial infarction, and stroke [6]. Consequently, randomized, controlled clinical trials using vitamin D supplementation have been carried out in patients with hypertension, myocardial infarction, stroke, and chronic heart failure secondary to left ventricular dysfunction [6,7]; although despite this recent clinical attention, the benefits of vitamin D supplementation in such diseases has not been well established.

In particular, the potential association between vitamin D and PH remains unclear with only a few reports suggesting that vitamin D deficiency is relevant to PH. For example, patients with vitamin D deficiency showed elevated systolic pulmonary artery pressure assessed by echocardiography [8]. Moreover, *in vitro* studies showed that vitamin D inhibited endothelin-induced vascular smooth muscle cell proliferation through suppression of cyclin-dependent kinase 2 activity [9], inhibited a tissue growth factor beta 1-related pro-fibrotic effect on lung fibroblasts and epithelial cells [10], and increased nitric oxide production through endothelial nitric oxide synthase activation [11].

Hence, this study aimed to 1) examine the relationship between serum 25(OH)D levels and clinical severity in PH patients, and 2) investigate whether dietary vitamin D supplementation could benefit the treatment of PH.

## Materials and methods

### Ethics statement

The animal studies were carried out in strict accordance with the recommendations in the Guide for the Care and Use of Laboratory Animals of the National Institutes of Health Guidelines. The Boards for Studies in Experimental Animals approved experimental procedures at Keio University School of Medicine and Nippon Veterinary and Life Science University, respectively. The human study was approved by the ethics committees of Keio University School of Medicine and Kyorin University School of Medicine, respectively. All patients provided written informed consent to participate.

### Patients and serum analysis

We measured serum 25(OH)D levels in blood collected from 41 PH patients with a mean pulmonary arterial pressure (PAP) > 30 mmHg and B-type natriuretic peptide (BNP) level > 20 pg/mL. Of this study group, 12 patients were diagnosed with idiopathic or heritable pulmonary arterial hypertension (PAH) and 29 patients with chronic thromboembolic pulmonary hypertension (CTEPH). Fasting blood samples were collected and centrifuged to obtain serum samples, which were stored at -80°C until analysis. Study patients were not necessarily treatment-naïve and some of them have been already diagnosed and treated by advanced therapies at the time of blood sampling. The serum 25(OH)D levels were measured by Oriental Yeast Co., Ltd (Japan) using radioimmunoassay. We analyzed the correlation between serum 25(OH)D levels and hemodynamic parameters assessed by right heart catheterization.

### Animal studies and vitamin D diets

Male rats weighing 90 to 110 g (CLEA) were used in our experiments. The rat PH model was generated as described previously [12,13]. In brief, 20 mg/kg of vascular endothelial growth factor inhibitor SU5416 (Abcam) suspended in CMC (0.5% [w/v] carboxymethylcellulose sodium, 0.9% [w/v] sodium chloride, 0.4% [v/v] polysorbate 80, and 0.9% [v/v] benzyl alcohol in deionized water) was subcutaneously injected at Day 0. The rats were housed in a hypoxic chamber (10% O_2_) maintained using a hypoxic air generator (TEIJIN) and monitored with an O_2_ analyzer (JIKO-255) for 3 weeks, and then re-exposed to normoxia for an additional 3 or 5 weeks. A purified AIN-93G containing 1,000 IU/kg of cholecalciferol (normal diet) and the modified AIN-93G containing 10,000 IU/kg of that (high vitamin D diet) were purchased from Oriental Yeast Co., Ltd (Japan). PH rats were divided into two groups and fed different dietary amounts of cholecalciferol on ad libitum.

### Survival analysis

Although Sprague-Dawley rats tolerate severe PH induced by SU5416 and hypoxia, it has been reported that Fischer 344 rats exhibit greater mortality [13]. PH rats induced by SU5416 and hypoxia have been widely used for the assessment in experiments of PH because of the recapitulation of the pathological features observed in patients with severe pulmonary arterial hypertension. Hence, 6-week-old male Fisher 344 rats with PH weighing 90 to 110 g (CLEA) were used in this study to evaluate the effect of dietary administration with high dose vitamin D on survival, defined as the time from SU5416 injection to death of the rats or 8 weeks after injection. This study was carried out according to the above protocol described in ‘Animal Studies and Vitamin D Diet’ sub-section (n = 20 rats per group). The PH rats were housed in a temperature regulated room with access to food and water ad libitum. Animal health and behavior were monitored every day by trained staffs, and all efforts were made to minimize suffering. Humane endpoints were not used in this study because survival of PH rats is mainly determined by RV dysfunction without any clear clinical, physiological and behavioral signs. In addition, it was essential to evaluate real mortality caused by RV dysfunction and the potential of vitamin D as a new drug for preventing RV remodeling and improving survival of PH in this study, taking into consideration the current recommendation of endpoints in pivotal clinical study [14], actual developmental process of recently approved therapies [1,2,15,16] and the latest experimental studies for a potential new treatment option in PH [17,18]. The Boards for Studies in Experimental Animals at Keio University School of Medicine and Nippon Veterinary and Life Science University specifically reviewed and approved the anticipated mortality in this study. As a result, 16 out of 20 rats in normal diet group and 10 out of 20 rats in high vitamin D diet found dead by 8 weeks after SU5416 injection.

### Hemodynamic measurements

At 3, 6 or 8 weeks after SU5416 injection into Sprague-Dawley rats, each rat was anesthetized using 2 to 3% isoflurane on a heat board with heart rate monitoring by electrocardiogram (n = 10–11 rats per group). A micro-tip catheter (Millar Instruments) was inserted into the right ventricle via the right jugular vein to measure right ventricular systolic pressure (RVSP). Hemodynamic measurements were analyzed using Lab Chart 8 (AD Instruments). After exsanguination, the RV was separated from the left ventricle plus septum (LV+S) for assessment of RV remodeling, and then snap-frozen in liquid nitrogen for storage at -80°C until further analysis.

### Real-time quantitative PCR

Total RNA was isolated from frozen RV samples using TRIzol Reagent (Thermo Fisher Scientific), and cDNA was synthesized from 200 ng of total RNA using the High Capacity cDNA Reverse Transcription Kit (Applied Biosystems) according to the manufacturer’s protocol. Quantitative mRNA expression was assessed by real-time quantitative PCR (qPCR) using the THUNDERBIRD SYBR qPCR Mix (TOYOBO). Samples were quantified using a hot start at 95°C for 15 minutes, 40 cycles performed at 94°C for 15 seconds, 57.5°C for 20 seconds, and 72°C for 20 seconds using the ViiA7 (Applied Biosystems). The data were analyzed by the delta delta CT method. The following primers were used: rat *GAPDH* (forward 5’-CTGCACCACCAACTGCTTAC-3’, reverse 5’-CAGAGGTGCCATCCAGAGTT-3’), rat *BNP* (forward 5’-GTCAGTCGCTTGGGCTGT-3’, reverse 5’-CAGAGCTGGGGAAAGAAGAG-3’). All qPCR data obtained were normalized to the housekeeping gene *GAPDH*.

### Blood and morphometric analyses

Blood samples were collected at 8 weeks after SU5416 injection into Sprague-Dawley rats. The serum 25(OH)D, calcium, and inorganic phosphorus levels were measured by Oriental Yeast Co., Ltd (Japan). The hearts were harvested and fixed in 10% formalin, embedded in paraffin, and then sectioned at 4-μm thickness. To evaluate gross morphology, short-axis heart sections were stained with hematoxylin and eosin (H&E). To quantify the cross-sectional area of RV cardiomyocytes, immunofluorescence staining of short-axis heart sections was performed with primary antibodies to α-actin (1:500, Santa Cruz) and Wheat Germ Agglutinin conjugated with Alexa Fluor 647 (1:100, Invitrogen). After overnight incubation, sections were washed and incubated with secondary antibodies, Alexa 594-conjugated goat anti-mouse IgG (1:400, Invitrogen). All sections were counterstained and mounted with Fluoromount-G, containing DAPI (eBioscience). The size of RV cardiomyocytes was assessed in 50 cardiomyocytes from an individual heart. The left lungs were harvested, inflated via the trachea with saline, and then fixed in 10% formalin. The lungs were embedded in paraffin, sectioned at 4-μm thickness, and stained with Elastica-van Gieson (EVG). To assess pulmonary vessel remodeling, percent medial wall thickness was analyzed in 10 small pulmonary vessels of 20 to100-μm diameters from an individual lung. Images were captured by light microscopy (BZ-9000; KEYENCE).

### Statistical analysis

All data are expressed as mean ± SEM. All statistical analyses were performed with Student’s *t* test or Fisher’s exact test for comparisons between two groups or one-way ANOVA followed by Scheffe’s method for multiple group comparisons. Correlation between 25(OH)D and hemodynamic parameters were determined by Spearman’s correlation coefficients. Comparisons of the time-course of parameters between the two groups were made by two-way ANOVA for repeated measures, followed by Newman-Keuls’ test. Survival was derived using the Kaplan-Meier method and compared using a log-rank test. All statistical analyses were performed with SPSS Statistics version 23.0 (IBM). A value of *P* < 0.05 was considered statistically significant.

## Results

### Correlation between serum 25(OH)D levels and hemodynamics in PH patients

Table 1 details characteristics of the 41 PH patients analyzed in this study. Overall, there were 28 females (68.3%), and the mean PAP, PVR, cardiac output, and six-minute-walk distance (6MWD) were 43.9 ± 1.5 mmHg, 11.2 ± 1.1 Wood units, 3.7 ± 0.2 L/min, and 369 ± 19 m, respectively. Compared to the CTEPH patients, PAH patients were more severe and were significantly younger (41.2 ± 5.1 years in PAH vs. 63.7 ± 2.6 years in CTEPH, *P* < 0.001), and had higher mean PAP (51.0 ± 2.9 mmHg in PAH vs. 40.9 ± 1.5 mmHg in CTEPH, *P* = 0.002) and PVR (16.2 ± 2.7 Wood units in PAH vs. 9.2 ± 0.8 mmHg in CTEPH, *P* = 0.002).

**Table 1:** Characteristics of patients with pulmonary hypertension (PH).

Variables	All patients (n = 41)	PAH (n = 12)	CTEPH (n = 29)	*P*-value of PAH vs. CTEPH
Gender, no. (%)				0.276
Male	13 (31.7%)	2 (16.7%)	11 (37.9%)	
Female	28 (68.3%)	10 (83.3%)	18 (62.1%)	
Age, years	57.3 ± 2.8	41.2 ± 5.1	63.7 ± 2.6	< 0.001*
Mean PAP, mmHg	43.9 ± 1.5	51.0 ± 2.9	40.9 ± 1.5	0.002*
Mean RAP, mmHg	6.1 ± 0.6	6.3 ± 1.0	6.0 ± 0.7	0.800
PCWP, mmHg	8.1 ± 0.4	8.0 ± 0.7	8.1 ±0.5	0.906
PVR, Wood units	11.2 ± 1.1	16.2 ± 2.7	9.2 ± 0.8	0.002*
Cardiac output, L/min	3.7 ± 0.2	3.3 ± 0.4	3.9 ± 0.2	0.171
Cardiac index, L/min/m^2^	2.4 ± 0.1	2.2 ± 0.3	2.5 ± 0.1	0.175
BNP, pg/mL	191 ± 35	204 ± 82	186 ± 36	0.816
6MWD, m	369 ± 19	383 ± 28	363 ± 25	0.651
Serum 25(OH)D levels, ng/mL	18.5 ± 1.0	13.1 ± 1.5	20.7 ± 1.0	< 0.001*

*Abbreviations*: PAH, idiopathic or heritable pulmonary arterial hypertension; CTEPH, chronic thromboembolic pulmonary hypertension; PAP, pulmonary arterial pressure; RAP, right atrial pressure; PCWP, pulmonary capillary wedge pressure; PVR, pulmonary vascular resistance; BNP, B-type natriuretic peptide; 6MWD, six-minute-walk distance; 25(OH)D, 25-hydroxyvitamin D. Data are mean ± SEM.

*P < 0.01.

**Fig 1:**
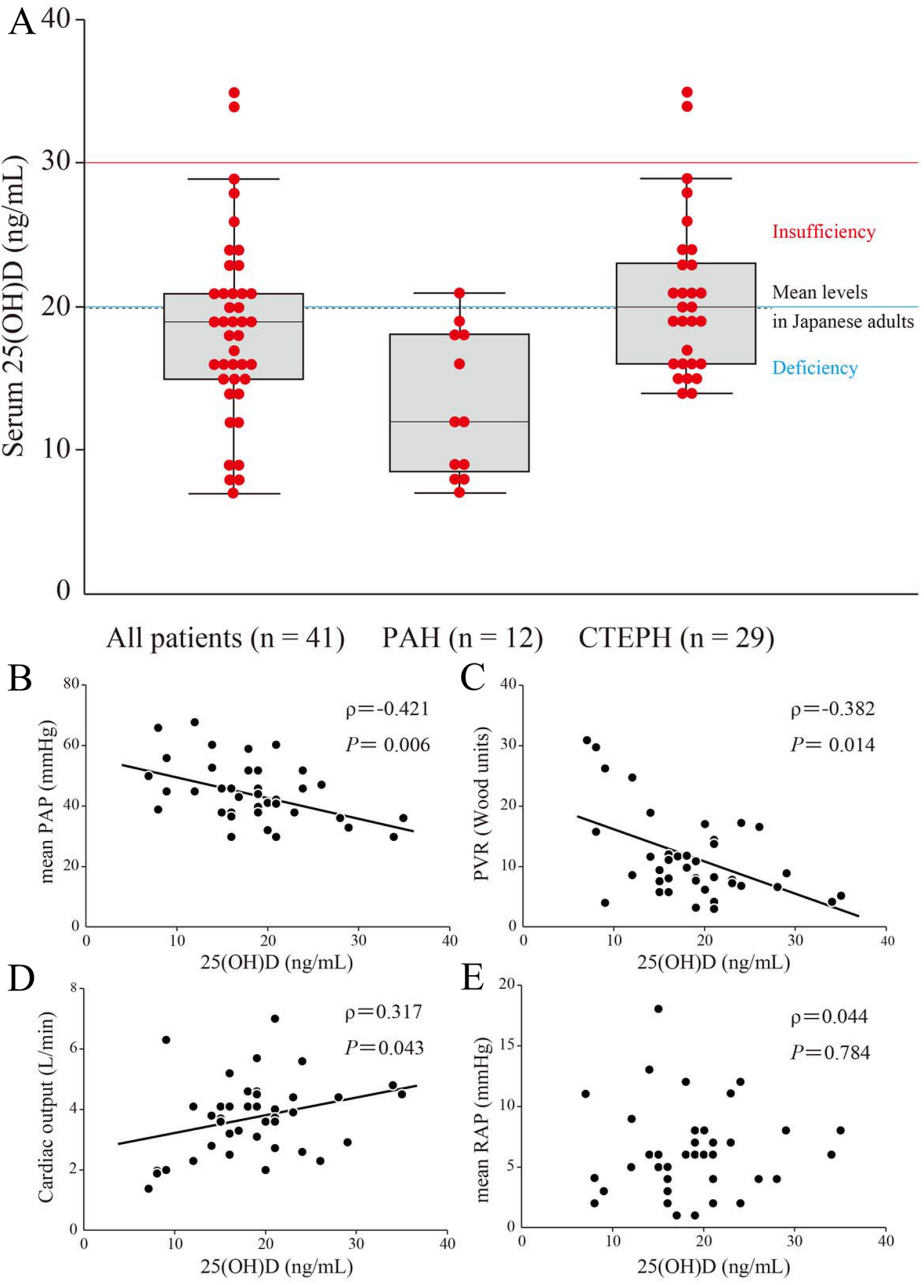
Relationship between serum 25-hydroxyvitamin D (25(OH)D) levels and hemodynamic parameters in patients with pulmonary hypertension (PH). Serum 25(OH)D levels and hemodynamic parameters were measured in 41 patients with PH, consisting of 12 patients with idiopathic or heritable pulmonary arterial hypertension (PAH) and 29 patients with chronic thromboembolic pulmonary hypertension (CTEPH). (A) Serum 25(OH)D levels in each PH classification. Serum 25(OH)D levels are shown as box plots, with bars denoting median values, boxes denoting interquartile ranges, and whiskers denoting ranges, excluding statistical outliers (●; >1.5 box lengths from either the 25th or 75th percentiles). Serum 25(OH)D levels of insufficiency (red line), deficiency (blue line), and the mean levels in Japanese adults (black broken line) are shown. (B)-(E) Correlations in 41 patients with PH were analyzed between 25(OH)D levels and mean pulmonary arterial pressure (PAP) (B), pulmonary vascular resistance (PVR) (C), cardiac output (D), and mean right atrial pressure (RAP) (E), respectively.

The reported mean 25(OH)D level in Japanese adults is 20.0 ng/mL (49.9 nmol/L) [19], compared to 18.5 ± 1.0 ng/mL for the PH patients studied herein (Table 1 and Fig 1A). Intriguingly, the PAH subgroup of patients had lower serum 25(OH)D levels than the CTEPH group (13.1 ± 1.5 ng/mL in PAH vs. 20.7 ± 1.0 ng/mL in CTEPH, *P* < 0.001) (Table 1 and Fig 1A). Vitamin D insufficiency and deficiency are defined as serum 25(OH)D levels of less than 30 ng/mL (75 nmol/L) and 20 ng/mL (50 nmol/L), respectively [5,20]. Hence, 39 out of the 41 PH patients (95.1%) were vitamin D insufficient, and 25 patients (61.0%) were deficient (Fig 1A).

Furthermore, we assessed the relationship between serum 25(OH)D levels and hemodynamics in the total PH patient group. Intriguingly, serum 25(OH)D levels showed a significant negative correlation with mean PAP (ρ = -0.421, *P* = 0.006) and PVR (ρ = -0.382, *P* = 0.014) (Fig 1B and 1C, and Table 2) and a significant positive correlation with cardiac output (ρ = 0.317, *P* = 0.043) (Fig 1D and Table 2), but no correlation with mean RAP (ρ = 0.044, *P* = 0.784) (Fig 1E and Table 2). Moreover, serum 25(OH)D levels showed a significant positive correlation with cardiac output (ρ = 0.609, *P* = 0.035) in the 12 PAH patients, despite the small number of patients (Table 2).

**Table 2:** Correlation between serum 25(OH)D levels and hemodynamic parameters in PH patients.

	All patients (n = 41)		PAH (n = 12)		CTEPH (n = 29)	
Variables	Correlation coefficient (ρ)	*P*-value	Correlation coefficient (ρ)	*P*-value	Correlation coefficient (ρ)	*P*-value
Mean PAP	-0.421	0.006†	-0.055	0.866	-0.292	0.124
Mean RAP	0.044	0.784	0.067	0.836	0.124	0.521
PCWP	-0.073	0.649	-0.411	0.184	0.047	0.807
PVR	-0.382	0.014*	-0.493	0.103	-0.250	0.190
Cardiac output	0.317	0.043*	0.609	0.035*	0.171	0.374
Cardiac index	0.213	0.207	0.405	0.192	0.041	0.847
BNP	-0.155	0.334	-0.338	0.283	-0.236	0.219
6MWD	0.230	0.165	0.306	0.359	0.298	0.131

Abbreviations are defined in Table 1.

*P < 0.05 in correlation analysis with serum 25(OH)D levels.

^†^*P* < 0.01 in correlation analysis with serum 25(OH)D levels.

### Improved survival with dietary vitamin D supplementation in PH rats

While we observed a significant relationship between serum 25(OH)D levels and hemodynamics in PH patients, it remains unclear whether lower serum 25(OH)D levels are caused by PH itself or affect the disease progression. Hence, we next investigated the effects of dietary vitamin D supplementation to elucidate the cause-result relationship between vitamin D and PH.

**Fig 2:**
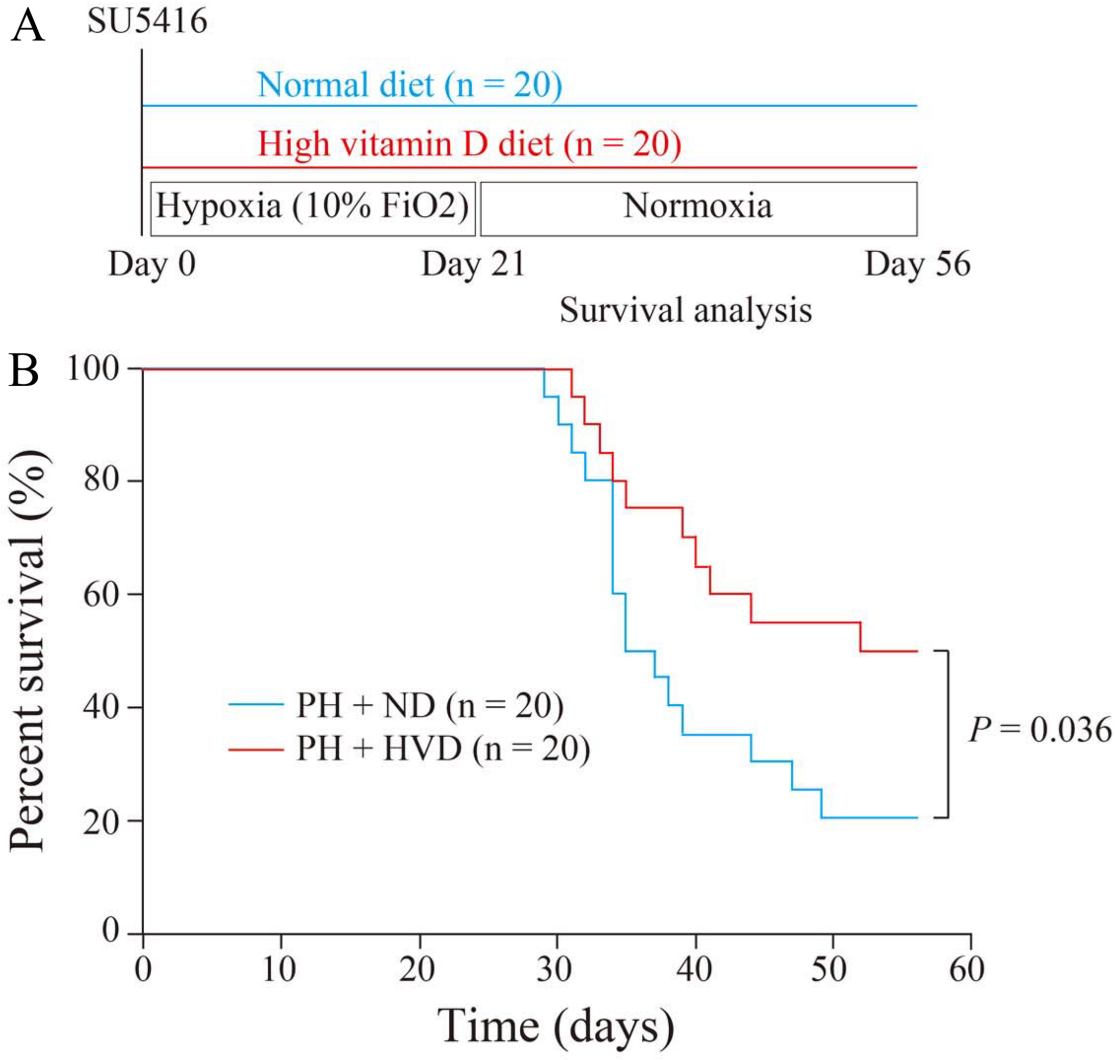
Survival benefit of dietary vitamin D supplementation in PH rats. (A) Experimental protocol for investigating the effects of dietary vitamin D supplementation on survival in Fisher 344 rats with PH. (B) Kaplan-Meier survival curves of PH rats receiving the normal diet (PH + ND group) and PH rats receiving the high vitamin D diet (PH + HVD group) (n = 20 rats per group).

Mortality is one of the most important and robust endpoints in the treatment of PH. Hence, we firstly assessed the effect of dietary vitamin D supplementation on survival in PH rats (Fig 2A). Notably, Kaplan-Meier survival curves demonstrated that PH rats receiving the high vitamin D diet (PH + HVD group) had a significantly higher survival rate compared to PH rats receiving the normal diet (PH + ND group) (50.0% in PH + HVD group vs. 20.0% in PH + ND group at 8 weeks after initiation of PH, *P* = 0.036) (Fig 2B).

### Effects of dietary vitamin D supplementation on prevention of right ventricular remodeling

Based on the beneficial effect of dietary vitamin D supplementation on survival in PH rats, we further investigated the effects of dietary vitamin D supplementation on the pulmonary vasculature and heart in PH rats.

**Fig 3:**
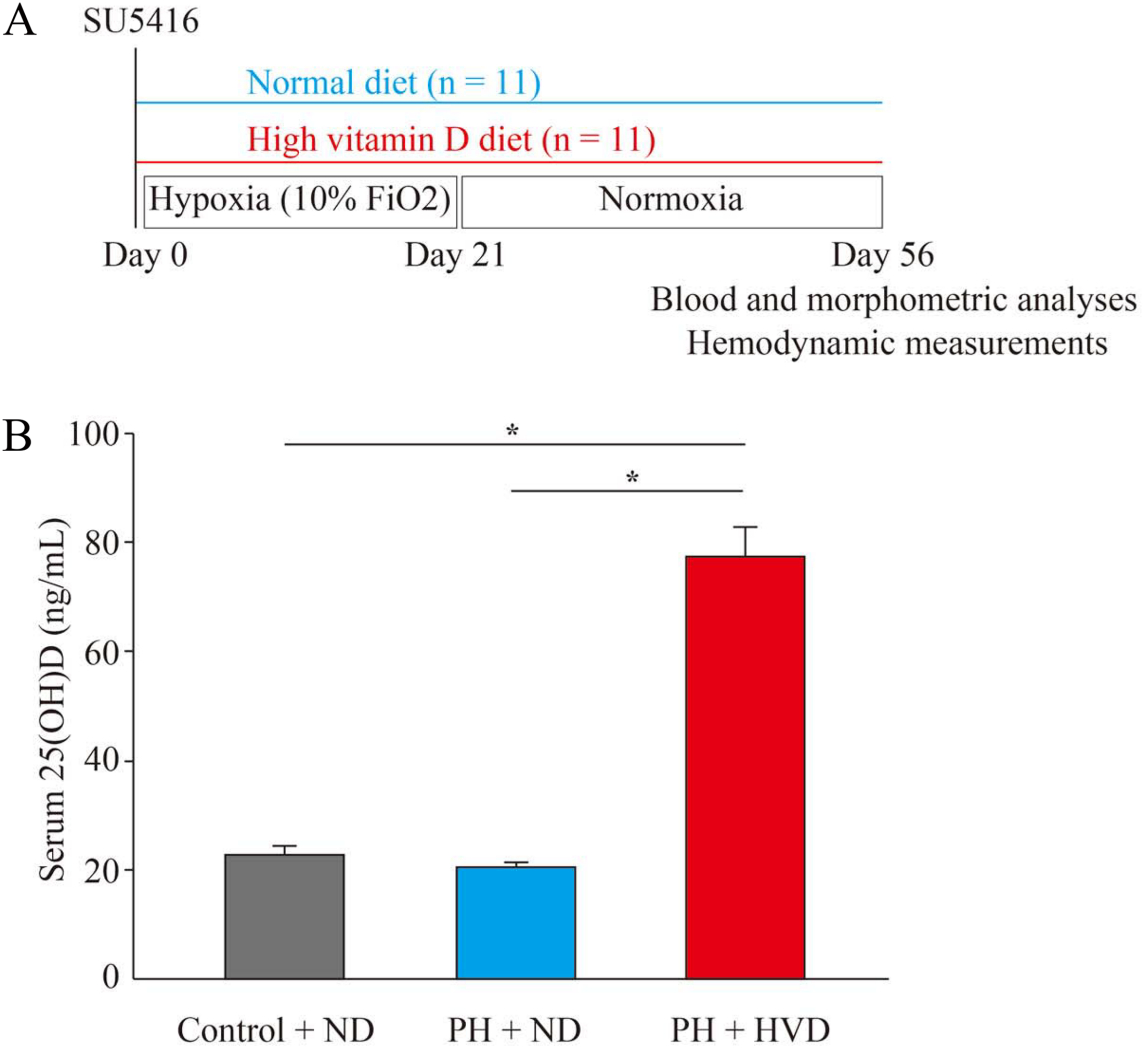
Experimental protocol and serum 25(OH)D levels in PH rats. (A) Experimental protocol for investigating the effects of dietary vitamin D supplementation in Sprague-Dawley rats with PH. (B) Serum 25(OH)D levels in control rats receiving the normal diet (control + ND group), PH rats receiving the normal diet (PH + ND group), or PH rats receiving the high vitamin D diet (PH + HVD group) (n = 8 rats per group). Data are mean ± SEM. *P < 0.01.

First, we demonstrated that dietary vitamin D supplementation affected the serum 25(OH)D levels in PH rats, which were generated by the experimental protocol shown in Fig 3A, with approximately 3.5-fold higher levels in PH rats of the PH + HVD group compared to control rats receiving the normal diet (control + ND group) and PH rats of PH + ND group (*P* < 0.001 in PH + HVD group vs. control + ND and PH +ND groups, respectively) (Fig 3B). This result confirmed that circulating 25(OH)D status was altered by dietary vitamin D intake. On the other hand, no differences were seen in serum calcium or inorganic phosphorus among the control + ND group, PH + ND group, and PH + HVD group (*P* > 0.05) (S1 File), nor were there any differences in body weight among these groups (Table 3).

**Table 3:** Physiological profiles of three experimental groups.

	Control + ND group (n = 10–11)	PH + ND group (n = 11)	PH + HVD group (n = 11)	*P*-value between PH + ND vs. PH + HVD groups
BW, g	487 ± 8	456 ± 15	476 ± 9	0.488
RV, g	0.26 ± 0.01	0.45 ± 0.03†	0.35 ± 0.02*	0.013‡
LV+S, g	0.88 ± 0.02	0.97 ± 0.02*	0.94 ± 0.02	0.679
RV/(LV+S)	0.30 ± 0.01	0.47 ± 0.03†	0.37 ± 0.02	0.025‡
RV/BW, mg/g	0.53 ± 0.02	1.03 ± 0.12†	0.73 ± 0.05	0.027‡
(LV+S)/BW, mg/g	1.80 ± 0.03	2.15 ± 0.09†	1.99 ± 0.03	0.118
HW/BW, mg/g	2.33 ± 0.02	3.18 ± 0.20†	2.72 ± 0.06	0.039‡
RVSP, mmHg	33 ± 1	92 ± 12†	72 ± 7†	0.243
HR, bpm	396 ± 9	342 ± 5†	342 ± 9†	1.000

*Abbreviations*: BW, body weight; RV, right ventricle; LV+S, left ventricle plus septum; RV/(LV+S), the ratio of RV weight to LV+S weight; RV/BW, the ratio of RV weight to BW; (LV+S)/BW, the ratio of LV+S weight to BW; HW/BW, the ratio of heart weight to BW; RVSP, right ventricular systolic pressure; HR, heart rate; bpm, beats per minute; control + ND group, control rats receiving normal diet; PH + ND group, rats with pulmonary hypertension receiving normal diet; PH + HVD group, rats with pulmonary hypertension receiving high vitamin D diet.

Data are mean ± SEM.

*P < 0.05 vs. Control + ND group;

^†^*P* < 0.01 vs. Control + ND group;

^‡^*P* < 0.05 between PH + ND group vs. PH + HVD group.

**Fig 4:**
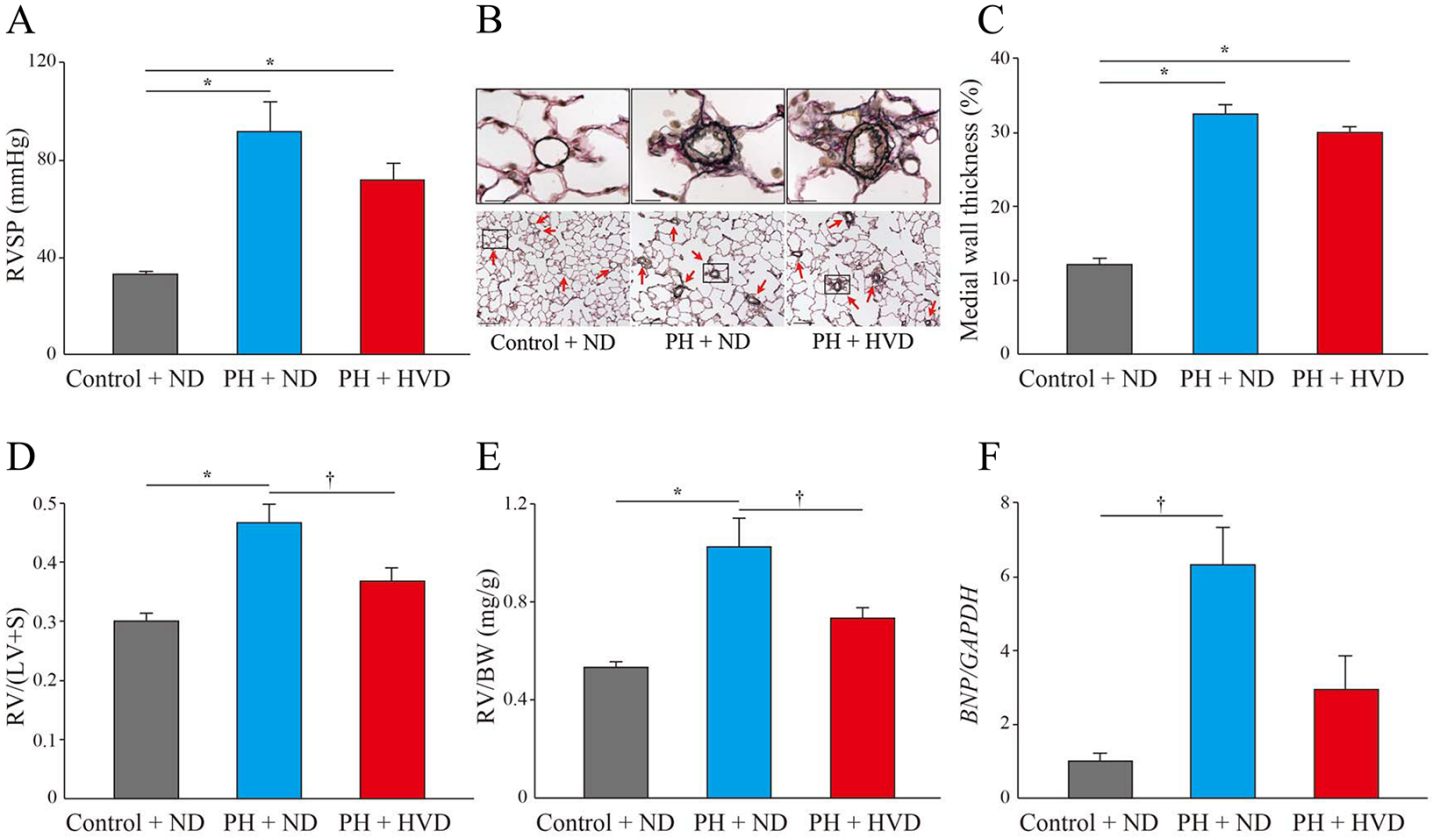
Effects of dietary vitamin D supplementation in PH rats. Assessments in Control + ND group, PH + ND group, and PH + HVD group (n = 10–11 rats per group). (A) Right ventricular systolic pressure (RVSP). (B) Representative Elastica-van Gieson (EVG) staining of lungs from rats in each experimental group. Red arrows indicate pulmonary arteries. Scale bars, 20 μm (above panels) and 100 μm (below panels). (C) Percent medial wall thickness in the small pulmonary arteries of 20–100 μm diameter (n = 4–10 rats per group). (D) The ratio of right ventricular weight to left ventricle plus septum weight [RV/(LV+S)]. (E) The ratio of right ventricular weight to body weight (RV/BW). (F) Relative expression of B-type natriuretic peptide (*BNP*) mRNA levels normalized to levels of glyceraldehyde 3-phosphate dehydrogenase (*GAPDH*) in the right ventricles of experimental rats. Expression was quantified by qPCR (n = 3–4 rats per group). Data are mean ± SEM. *P < 0.01, †*P* < 0.05.

Second, at 8 weeks, RVSP was significantly increased in the PH + ND group compared to control + ND rats, but there was no significant difference in RVSP between the PH + ND and PH + HVD groups (92 ± 12 mmHg in PH + ND group vs. 72 ± 7 mmHg in PH + HVD group, *P* = 0.243) (Fig 4A). Histological and quantitative analyses of lungs showed marked pulmonary vascular remodeling and significantly increased percent medial wall thickness in the PH + ND group compared to the control + ND group, but again there was no significant difference between PH + ND and PH + HVD groups (medial wall thickness, 32.4 ± 1.3% in PH + ND group vs. 29.9 ± 0.8% in PH + HVD group, *P* = 0.179) (Fig 4B and 4C).

**Fig 5:**
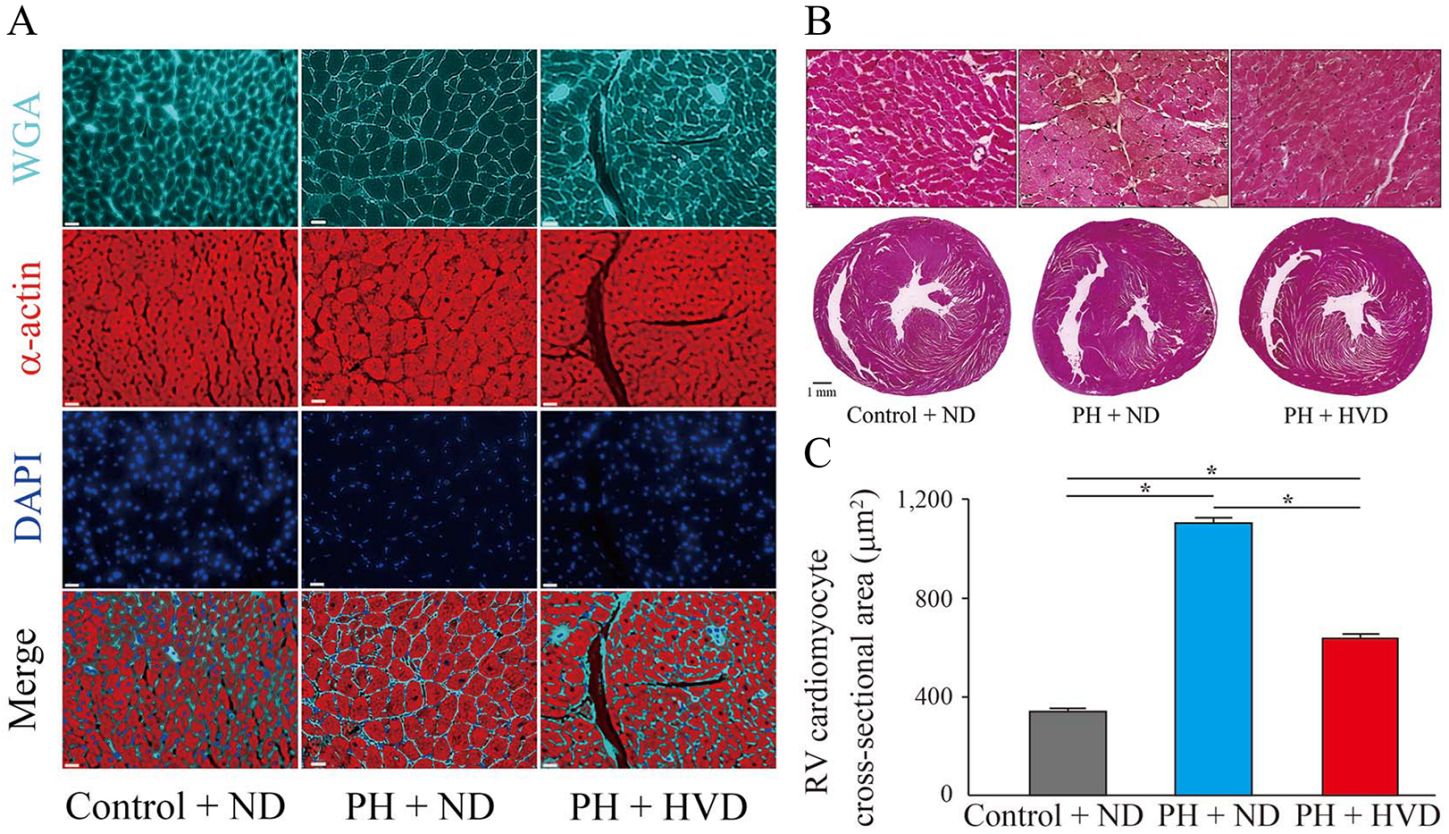
Inhibition of hypertrophy in right ventricular (RV) cardiomyocytes by dietary vitamin D supplementation in PH rats. (A) and (B), Representative images of hearts from rats in each experimental group. (A), Wheat germ agglutinin (WGA), α-actin, and 4’,6-diamidino-2-phenylindole (DAPI) staining of RV cross sections. Scale bars, 20 μm. (B) Hematoxylin and eosin (H&E) staining. Above panels show magnified images of cardiomyocytes in RV wall. Scale bars, 20 μm (above panels) and 1 mm (below panels). (C) Quantification of RV cardiomyocyte with cross-sectional area stained (n = 5 rats per group). Data are mean ± SEM. *P < 0.01.

Third, at 8 weeks, RV remodeling determined by the ratio of RV weight to LV+S weight [RV/(LV+S)] and RV weight to body weight (RV/BW) was evident in the PH + ND group, whereas it was significantly attenuated in the PH + HVD group [RV/(LV+S), 0.47 ± 0.03 in PH + ND group vs. 0.37 ± 0.02 in PH + HVD group, *P* = 0.025; RV/BW, 1.03 ± 0.12 mg/g in PH + ND group vs. 0.73 ± 0.05 mg/g in PH + HVD group, *P* = 0.027] (Fig 4D and 4E). In addition, compared to the PH + ND group, RV weight and the ratio of total heart weight to body weight (HW/BW) were also significantly decreased in the PH + HVD group (RV weight, 0.45 ± 0.03 g in PH + ND group vs. 0.35 ± 0.02 g in PH + HVD group, *P* = 0.013; HW/BW, 3.18 ± 0.20 mg/g in PH + ND group vs. 2.72 ± 0.06 mg/g in PH + HVD group, *P* = 0.039) (Table 3). Furthermore, we observed a significant increase in *BNP* mRNA expression in the RV of PH + ND rats compared to the control + ND group, whereas it was attenuated in the PH + HVD group (Fig 4F). Consistent with the above observations, representative histological analyses of hearts showed a marked increase in cross-sectional area of individual RV cardiomyocytes in the PH + ND group compared to the control + ND group, whereas these areas were significantly attenuated in the PH + HVD group (1,102 ± 23 μm^2^ in PH + ND group vs. 630 ± 19 μm^2^ in PH + HVD group, *P* < 0.001) (Fig 5).

**Fig 6:**
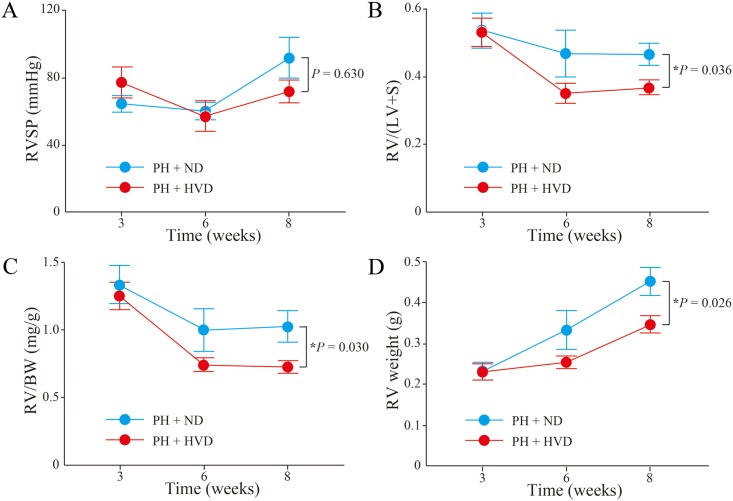
Time-course changes of PH and RV remodeling in PH rats. Time-course data were compared between PH + ND group and PH + HVD group (n = 5–11 rats per group). (A) RV systolic pressure (RVSP). (B) The ratio of right ventricular weight to left ventricle plus septum weight [RV/(LV+S)]. (C) The ratio of right ventricular weight to body weight (RV/BW). (D) RV weight. Data are mean ± SEM. *P < 0.05.

Finally, we further assessed the time-course changes of pulmonary hypertension and RV remodeling at 3, 6 and 8 weeks. As a result, there was no significant time-course change in RVSP between the PH + ND and PH + HVD groups (*P* = 0.630) (Fig 6A). Meanwhile, a significant time-course inhibition of RV remodeling was observed in PH + HVD group compared to the PH + ND group [RV/(LV+S), *P* = 0.036; RV/BW, *P* = 0.030; and RV weight, *P* = 0.026] (Fig 6B, 6C and 6D).

## Discussion

In the present study, we demonstrated that 1) serum 25(OH)D levels were significantly correlated with hemodynamics in PH patients, 2) dietary vitamin D supplementation significantly improved survival in PH rats, and 3) dietary vitamin D supplementation significantly attenuated RV remodeling in PH rats, although PH, per se, did not apparently change with vitamin D supplementation.

Advanced vasodilator therapies introduced for the management of PH have improved the clinical outcomes of patients over the last two decades [1,2]. In addition, many experimental studies have been carried out based on new concepts targeting cell proliferation, inflammation, fibrosis, and/or metabolism. Unfortunately, despite such clinical advances and even if such studies show positive effects for experimental PH, there are no approved drugs other than vasodilators, and concerns remain regarding their possible side effects. Thus, having only the mechanism of vasodilation as treatment is a limitation for PH therapy, and for some patients PH remains progressive and fatal, highlighting the unmet medical need for new approaches in PH management.

In this study, we first confirmed that the mean serum 25(OH)D level in PH patients was lower than that in the Japanese adult population [19], and that this difference was more marked in our PAH patient subgroup. Furthermore, 95.1% of PH patients had vitamin D insufficiency, and 61.0% had vitamin D deficiency, and we found statistically significant relationships between serum 25(OH)D levels and hemodynamics assessed by right heart catheterization in our PH patient cohort. Moreover, serum 25(OH)D levels were significantly correlated with cardiac output in PAH patients, who showed more severe disease than the CTEPH patients examined in this study, although the levels were not correlated with mean PAP or PVR, suggesting that serum 25(OH)D level reflects clinical severity in PH patients. Importantly, this demonstrated correlation between serum 25(OH)D levels and cardiac output in PAH patients infers that vitamin D has a key role in the pathogenesis of PH and its related RV dysfunction, because cardiac output is determined by RV function and regulates prognosis in PH.

To clarify whether the lower serum 25(OH)D levels are caused by the PH itself or instead affect disease progression, we administered vitamin D to experimental PH rats to examine the cause-result relationship, using a dose based on previous findings [21]. Survival, which is mainly determined by RV dysfunction in PH, was significantly improved in the PH rats administered dietary vitamin D supplementation, although the mechanism underlying this favorable effect remained unclear. Hence, we further examined the effects of dietary vitamin D supplementation on pulmonary vasculature and RV remodeling in PH rats. Intriguingly, histological analysis and the measurement of cross-sectional area of RV cardiomyocytes demonstrated that dietary vitamin D supplementation significantly suppressed RV remodeling in PH rats, although the medial wall thickness of pulmonary arteries was not affected by vitamin D supplementation, which was consistent with no significant improvement of RVSP. Furthermore, the assessment of time-course changes showed no significant differences in RVSP during the study period and a significant improvement in RV remodeling. These results strongly suggest that vitamin D supplementation could directly inhibit the RV remodeling via regulation of cardiomyocyte hypertrophy, without changes to PH. Recently, the VINDICATE study demonstrated that vitamin D supplementation could improve left ventricular function in patients with chronic heart failure [7]. Hence, our findings raise the possibility that vitamin D can affect cardiomyocytes in both ventricles, leading to overall functional improvement. Combined with our result that lower serum 25(OH)D levels correlate with more severe hemodynamics in PH patients, an insufficient intake of vitamin D might potentially accelerate RV dysfunction in PH. Hence, combination therapy using vasodilators and vitamin D might induce more favorable outcomes in PH patients by more effectively improving both PAP and RV remodeling. Moreover, although there are no available specific drug therapies for PH other than PAH and CTEPH, the findings in this study suggest that vitamin D can be a new treatment option for broader types of PH including that due to left heart disease, lung disease and/or hypoxia, or with unclear and/or multifactorial mechanisms, at least in part through the inhibition of RV remodeling.

There are some limitations in this study. First, regarding to the human sample data of serum 25(OH)D levels, we cannot exclude the influence of potential confounding factors including age, sex, sun exposure, dietary intake and use of vitamin D, PAH-specific drugs that had been already administered at the time of blood sampling, or co-morbidities, and a possibility that lower serum 25(OH)D levels might be a consequence of the disease with less outdoor activities and therefore lower 25(OH)D levels in patients with more advanced disease. Second, because high-dose vitamin D was administered from the baseline of SU5416 injection and start of hypoxic condition to the end of the study, the best timing to start vitamin D supplementation in the clinical setting remains unclear. Third, we used doses containing 1,000 IU/kg and 10,000 IU/kg of cholecalciferol as normal and high doses of vitamin D, respectively, and optimal dosage to achieve clinical benefit will necessarily need further controlled studies. Fourth, vitamin D exhibits extensive pleiotropic effects clinically via diverse signaling pathways under pathological conditions. Further experimental studies are thus needed to explore the mechanisms underlying the findings in this study.

In conclusion, our findings support a periodic assessment of serum 25(OH)D levels and clinical evaluation of the necessity of vitamin D supplementation in PH patients as a preventive measure of disease progression and/or an additional treatment option. Vitamin D has been widely used for the treatment of various pathological conditions [22], and the safety profile has been well established. Hence, our findings could advance the potential of vitamin D as a new and safe drug for preventing RV remodeling and improving survival in PH patients.

## Supporting information

S1 File Data tables. (XLSX)
